# MicroRNA 322-5p reduced neuronal inflammation via the TLR4/TRAF6/NF-κB axis in a rat epilepsy model

**DOI:** 10.1515/med-2022-0485

**Published:** 2022-05-13

**Authors:** Qin Zhou, Qiong Wang, Baomei He, Haibo Kong, Huanjun Luo, Xiaowei Wang, Wenlan Wang

**Affiliations:** Department of Pediatrics, Zhejiang Provincial People’s Hospital, People’s Hospital of Hangzhou Medical College, Hangzhou 310014, Zhejiang Province, China; Department of Pediatrics, The Second Affiliated Hospital of Jiaxing University, Jiaxing 314000, Zhejiang Province, China; School of Clinical Medicine, Bengbu Medical College, Bengbu 233030, Anhui Province, China

**Keywords:** epilepsy, inflammation, miR-322-5p, TRAF6, NF-κB, apoptosis

## Abstract

This study aimed to determine whether microRNA-322-5p regulates seizure and seizure damage by targeting the TLR4/TRAF6/NF-κB-associated inflammatory signaling pathway. In a pilocarpine-induced epileptic rat model, the expressions of miR-322-5p, TLR4, NF-κB, TRAF6, IRF5, IL-1β, and GABA were assessed by a quantitative polymerase chain reaction and western blotting. Tunel detects hippocampal neuron apoptosis. The results showed that the expression of miR-322-5p significantly decreased in status epilepticus (SE) rats. The reduction of miR-322-5p was accompanied by increased levels of pro-inflammatory cytokines, an increased NF-κB expression, and reduced γ-aminobutyric acid (GABA) levels. Exogenous miR-322-5p reduced the expression of inflammatory molecules and increased the GABA levels in SE rats, and also reduced hippocampal neuronal cell apoptosis caused by epilepsy. In conclusion, the miR-322-5p significantly inhibited the TLR4/TRAF6/NF-κB-associated inflammation and reduced neuronal apoptosis, suggesting that its induction may be of potential interest for novel antiseizure medications.

## 1 Introduction

Status epilepticus (SE) in childhood is a medical emergency that may lead to permanent brain damage, neurological sequelae, or death. The incidence of SE is 18–23 per 100,000 children per year and the most common cause is febrile seizure. In children with epilepsy, most SE occurs at or prior to the diagnosis of epilepsy. The goal of therapy is the rapid termination of seizure activity, since appropriate and timely therapy of SE reduces the associated mortality and morbidity [1]. However, patients still receive inadequate treatment for a variety of reasons.

Emerging evidence indicates that SE occurs due to the failure of seizure termination [2]. Different biological processes have been proposed to lead to seizure termination including release of inflammatory peptides and decreased activity of γ-aminobutyric acid (GABA) [3]. The Toll-like receptor (TLR) family is constitutively expressed by brain cells and the signaling activation in brain inflammation has been well documented [4]. TLR4 stimulates nuclear factor kappa-B (NF-κB) through tumor necrosis factor receptor-associated factor 6 (TRAF6) and interferon regulatory factor 5 (IRF5), thereby activating inflammatory pathways [5]. TLR4 also modulates GABAergic synaptic activities [6]. However, the involvement of TLR4 signaling in epilepsy has not been fully elucidated.

MicroRNAs (or miRNAs) are a class of non-coding RNAs that play important roles in regulating gene expression and may contribute to the development of epileptogenesis [7]. A previous study reported specific upregulated and downregulated miRNAs in a rat model of SE [8]. The miR-322-5p is involved in the TLR4 signaling pathway and is decreased in the rat brain during SE. In the current study, we aimed to demonstrate the relationship among miR-322-5p, the TLR4/TRAF6/NF-κB signaling pathway, and apoptosis, speculating on their potential involvement as a target for new antiseizure medications.

## 2 Materials and methods

### 2.1 Pilocarpine-induced epileptic rat model

Male Sprague Dawley rats (3 weeks old) were purchased from SLAC (Shanghai, People’s Republic of China) and maintained at a constant temperature of 23 ± 2°C, with a 12-h light/dark cycle (light on from 08:00 to 20:00 h) and food and water ad libitum. All procedures were conducted according to the regulations set up by the Animal Experiment Facility and approved by the Experimental Animal Ethical Committee of Zhejiang Provincial People’s Hospital. A total of 72 rats were used to develop an epilepsy model through lithium chloride injection (3 mEq/kg, intraperitoneal [i.p.]), followed by an injection of methyl scopolamine bromide (1 mg/kg, 18 h later). After 30 min, the rats were injected with pilocarpine hydrochloride (100 mg/kg, i.p.) and then monitored to detect seizure activity within the next 2 h. Convulsive scores were given based on Racine’s scores for seizures as follows: 0, no response; 1, face and vibrissae twitching, ear rubbing on forepaws or chewing; 2, nodding of the head or unilateral limb clonus; 3, limb clonus or mild convulsions; 4, rearing with bilateral forelimb clonus, tail hypertension, lockjaw, or whole-body convulsions; 5, body convulsions with rearing or collapsing body with rigidity. The onset of SE was defined as reaching scores 4 or 5 [9]. Chloral hydrate (400 mg/kg, i.p.) and atropine (1 mg/kg, i.p.) were subsequently injected 30 min post onset of SE to attenuate seizure activity [10].

### 2.2 Animals and interventions

The rats were divided and used for two experiments: (1) the expressions of miR-322-5p, TLR4, TRAF6, the NF-κB subunit RelA, IRF5, and GABA were evaluated at different time points (1 week, 2 weeks, 3 weeks, and 4 weeks, *n* = 8 in each group) and (2) the effects of exogenous miR-322-5p were evaluated. Rats were divided into control (C), epilepsy (EP), epilepsy + vehicle (EP + V), and epilepsy + miR-322-5p (EP + miR) intervention group (*n* = 8 in each group). MiR-322-5p intervention group received 10 µL miR-322-5p (50 µmol/L) 30 min after epilepsy modeling for 3 consecutive days. The primer sequences were designed and synthesized by Shanghai GeneChem Co., Ltd. (Shanghai, China) (Table 1). Following each injection, the needle was left in place for 5 min to allow complete diffusion of the injected material [11]. The SE + vehicle group received 10 µL vehicle. The control group and the epilepsy group received the same volume of normal saline for microinjection. One week after treatment, all animals were killed and specimens were kept. The left brain was quickly removed on ice to make homogenate and stored in liquid nitrogen. The right hippocampal tissues were collected and fixed in 4% paraformaldehyde for 2 h, embedded in paraffin, and then sectioned and stained.

**Table 1:** Primer sequences for real-time PCR

Gene name	Forward	Reverse
miR-322-5p	AGCGTGCTGTGCGTGTGAC	CAGTGCAGGGTCCGAGGTATT
TLR4	CTACCTCGAGTGGGAGGACA	TGCTACTTCCTTGTGCCCTG
GABA	AATGGGCGGATTGGTGTC	TCATCTTGGGAGGGCTGT
IL-1β	CACCTCTCAAGCAGAGCACAG	GGGTTCCATGGTGAAGTCAAC
NF-kB (RelA)	AATTGCCCCGGCAT	TCCCGTAACCGCGTA
TRAF6	CAGTCCCCTGCACATT	GAGGAGGCATCGCAT
GAPDH	AGCCACATCGCTCAGACA	TGGACTCCACGACGTACT

### 2.3 Quantitative real-time PCR

The expressions of inflammatory genes and miR-322-5p were measured by quantitative real-time PCR. Total RNAs were extracted by TRIzol reagent (Takara, China). The cDNAs were transcribed into mRNA transcripts and the quantitative PCR was performed using a LightCycler 96 system (Hoffman-La Roche Ltd., Basel, Switzerland), with the following parameters: initial denaturation at 95°C for 5 min, denaturation at 95°C for 30 s, annealing at 58°C for 30 s, and 30 cycles of extension at 72°C for 30 s. The expressions of the genes of interest were determined by the 2^−ΔΔCT^ method, with U6 and GAPDH serving as endogenous controls. All primer sequences are displayed in Table 1.

### 2.4 Western blot

Proteins from the brain homogenates were extracted using RIPA buffer (Beyotime Biotechnology, China) supplemented with protease inhibitors (ComWin Biotech, China). The concentration of the protein extracts was determined using the BCA Protein Assay Kit (Solarbio, China). Each sample (40 µg) was loaded into a 10% sodium dodecyl sulfate-polyacrylamide gel, electrophoresed, and transferred to polyvinylidene fluoride (PVDF) membranes (GE Healthcare Life, China). PVDF membranes were then blocked with 5% BSA for 1 h at room temperature and incubated with primary antibodies. The primary antibodies were anti-TLR4 (ab13556), anti-TRAF6 (ab137452), anti-RelA (ab76302), anti-IL-1β (ab283818), anti-GABA (ab185205), and anti-GAPDH (ab8245). They were all purchased from Cell Signaling Technology (Abcam, UK). All membranes were subsequently probed with HRP-conjugated secondary antibodies (Sigma-Aldrich) for 1 h at room temperature (1:6,000 dilution). An ECL kit (Biosharp, China) was used to detect the protein-antibody signal.

### 2.5 Tunel assay

Tunel assay was used to detect the apoptosis of hippocampal neurons following the instructions given in the kit (Servicebio, China). After they were dewaxed and rehydrated, the paraffin sections were digested with proteinase K (Servicebio, China). Then equilibration buffer was added dropwise, incubated for 20 min, and the buffer was discarded. Appropriate amount of TDT enzyme, dUTP, and buffer was added dropwise in a ratio of 1:5:50 and incubated at 37°C for 2 h. DAPI (Servicebio, China) counterstains the nucleus. After performing the standard procedures as described in the instructions of the assay kit, the images were taken under fluorescence microscope (Nikon Eclipse C1, Japan). The blue color indicates normal cell nucleus and green indicates positive apoptotic cell nucleus. The apoptotic rate = tunel-positive cell numbers/total cell numbers × 100%.

### 2.6 Statistical analysis

All the statistical analyses were processed with SPSS 17.0 (SPSS Inc., Chicago, IL, USA). Data are presented as mean ± standard deviation. The comparison of data between the two groups was carried out by student’s *t*-test, and one-way analysis of variance was used to analyze the difference among multiple groups. *P*-value < 0.05 was considered statistically significant.

## 3 Results

### 3.1 SE rats showed an elevated TLR4/NF-κB expression and a decreased GABA level

**Figure 1:**
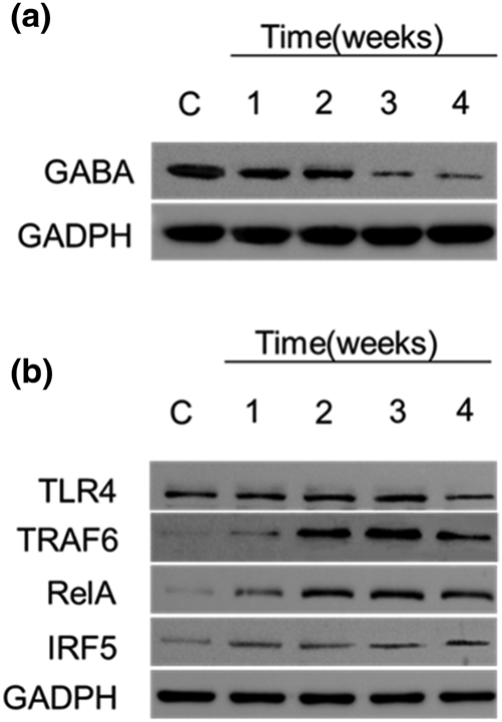
The TLR4/TRAF6/NF-kB signaling pathway in status epilepticus rats. Notes: (a) GABA and GAPDH levels in status epilepticus rats. (b) Western blot of rat brain revealed that the increased expression of TLR4, TRAF6, IRF5, and RelA post SE induction.

In the pilocarpine-induced epileptic rat model, the level of GABA was significantly reduced compared to controls (Figure 1a). The expression of TLR4, TRAF6, RelA (NF-κB subunit), and IRF5 increased in a time-dependent manner (Figure 1b), suggesting that inflammation occurred in the brain of SE rats.

### 3.2 miR-322-5p was downregulated in SE rats

**Figure 2:**
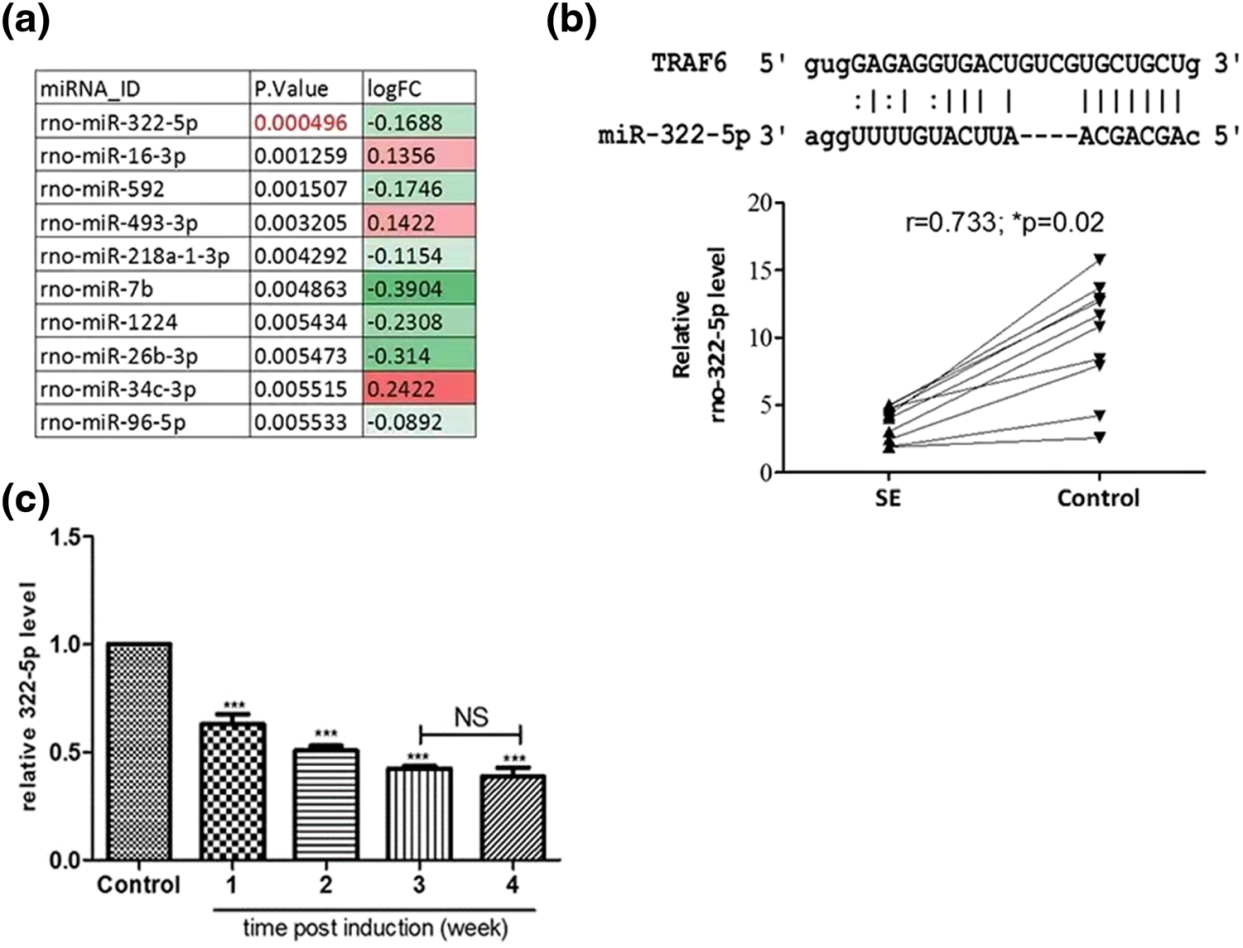
The expression of miR-322-5p. Notes: (a) miR-322-5p was identified as one of the most decreased microRNA species post SE induction in rats’ brain (GSE49850). (b) Insert demonstrates the binding sequences of miR-322-5p and the 3′-UTR of TRAF6. Lower the panel, a relatively strong negative correlation was identified between the expression of miR-322-5p and TRAF6 mRNA in patients with intractable epilepsy. (c) Real-time PCR analysis of mRNA isolated from SE rat brains indicated a significantly decreased miR-322-5p in the SE rats post induction. ***p < 0.001 compared with control.

The expression of miR-322-5p was decreased in SE rats (Figure 2a). According to several target gene prediction tools (miRmap, TargetScan, and PITA), miR-322-5p binds to the 3′-UTR of TRAF6, one of the key TLR4/NF-κB signaling components (upper panel, Figure 2b). A further evaluation indicated a negative relationship between the expression of miR-322-5p and the incidence of SE (lower panel, Figure 2b). One week after SE induction by pilocarpine, the level of miR-322-5p reduced and continued to decrease during the subsequent 3 weeks (Figure 2c).

### 3.3 Exogenous miR-322-5p reduced inflammation in SE rats

**Figure 3:**
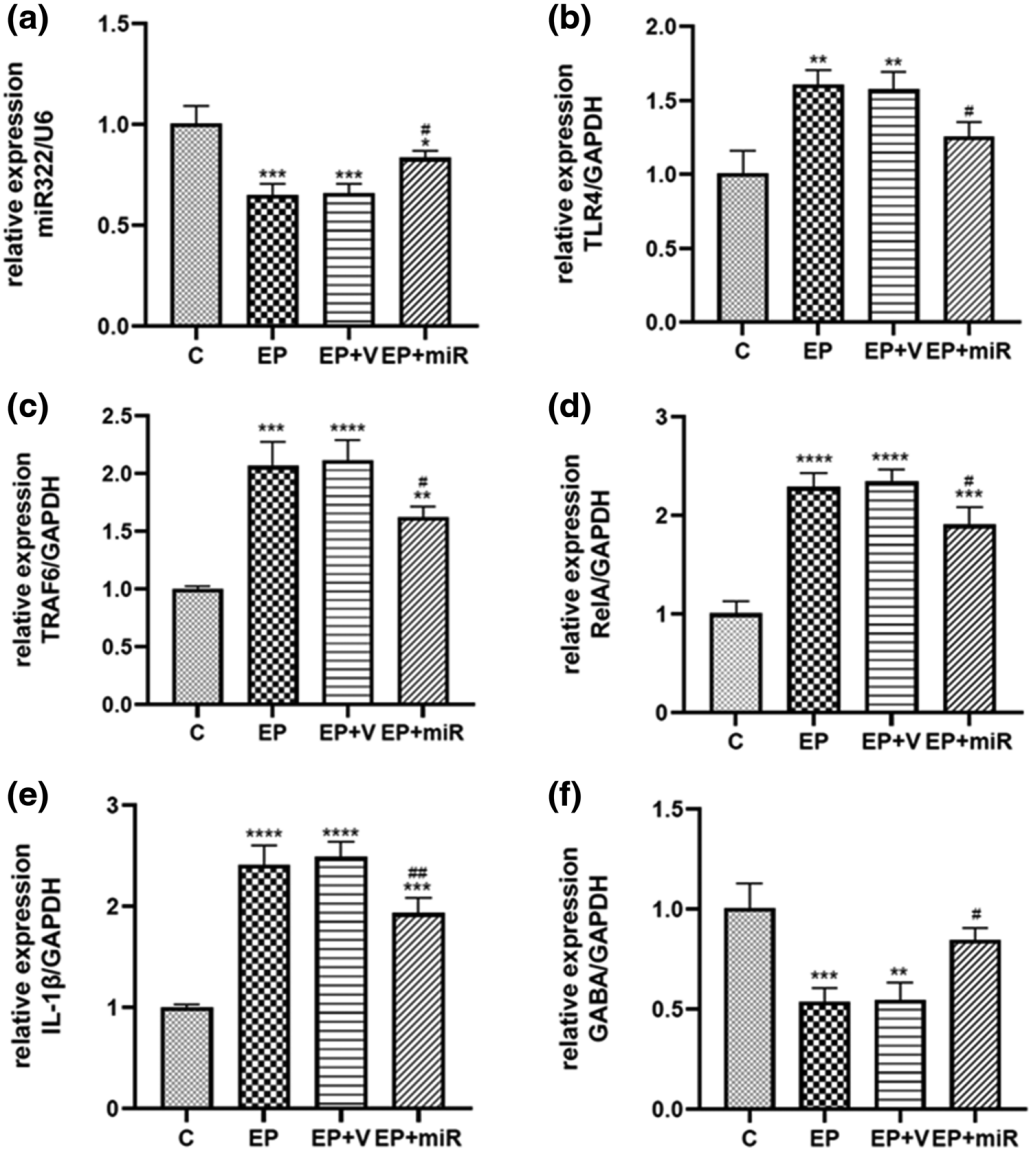
qRT-PCR analysis of the inflammatory markers and miR-322-5p. Notes: (a) The miR-322-5p-treated rats showed a higher miR-322-5p level than EP + V group. (b) TLR4 levels decreased after miR-322-5p injection. (c) TRAF6 levels decreased after miR-322-5p injection. (d) RelA levels decreased after miR-322-5p injection. (e) IL-1β levels decreased after miR-322-5p injection. (f) GABA levels increased after miR-322-5p injection. *p < 0.05, **p < 0.01, ***p < 0.001, ****p < 0.0001 compared with control; #*p* < 0.05, ##*p* < 0.01 compared with EP + V group. Abbreviations: C: control, EP: epilepsy, EP + V: epilepsy + vehicle, EP + miR: epilepsy + miR-322-5p intervention group.

**Figure 4:**
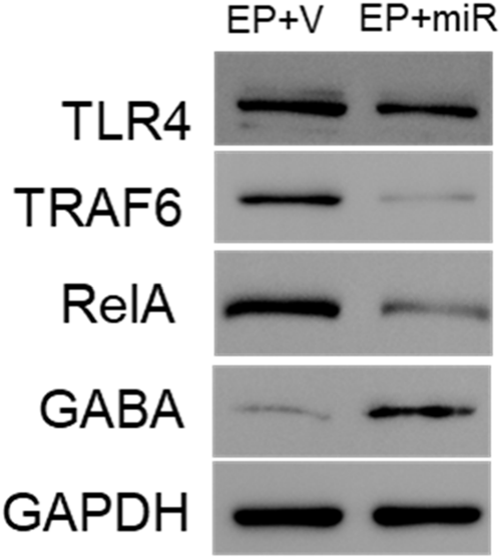
Western blot detected the changes of inflammatory markers after injection of exogenous miR-322-5p. Abbreviations: EP + V: epilepsy + vehicle, EP + miR: epilepsy + miR-322-5p intervention group.

Compared with normal control, TLR4, TRAF6, RelA, and IL-1β expressions were significantly upregulated after SE (Figure 3b–e). The level of miR-322-5p was elevated in the brain of miR-322-5p-injected rats compared to the EP + V group (Figure 3a). The rat brain tissues injected with exogenous miR-322-5p after SE induction showed a lower level of inflammation, as reflected by the reduced expression of TLR4, TRAF6, RelA, and IL-1β (Figure 3b–e). In contrast, GABA was reduced in epilepsy model, but upregulated after injecting miR-322-5p (Figure 3f). The western blot of brain homogenates showed lower expressions of inflammatory markers after being injected miR-322-5p, including TLR4, TRAF6, RelA, and IL-1β, and increased expressions of GABA (Figure 4).

### 3.4 Exogenous miR-322-5p inhibited apoptosis in SE rats

**Figure 5:**
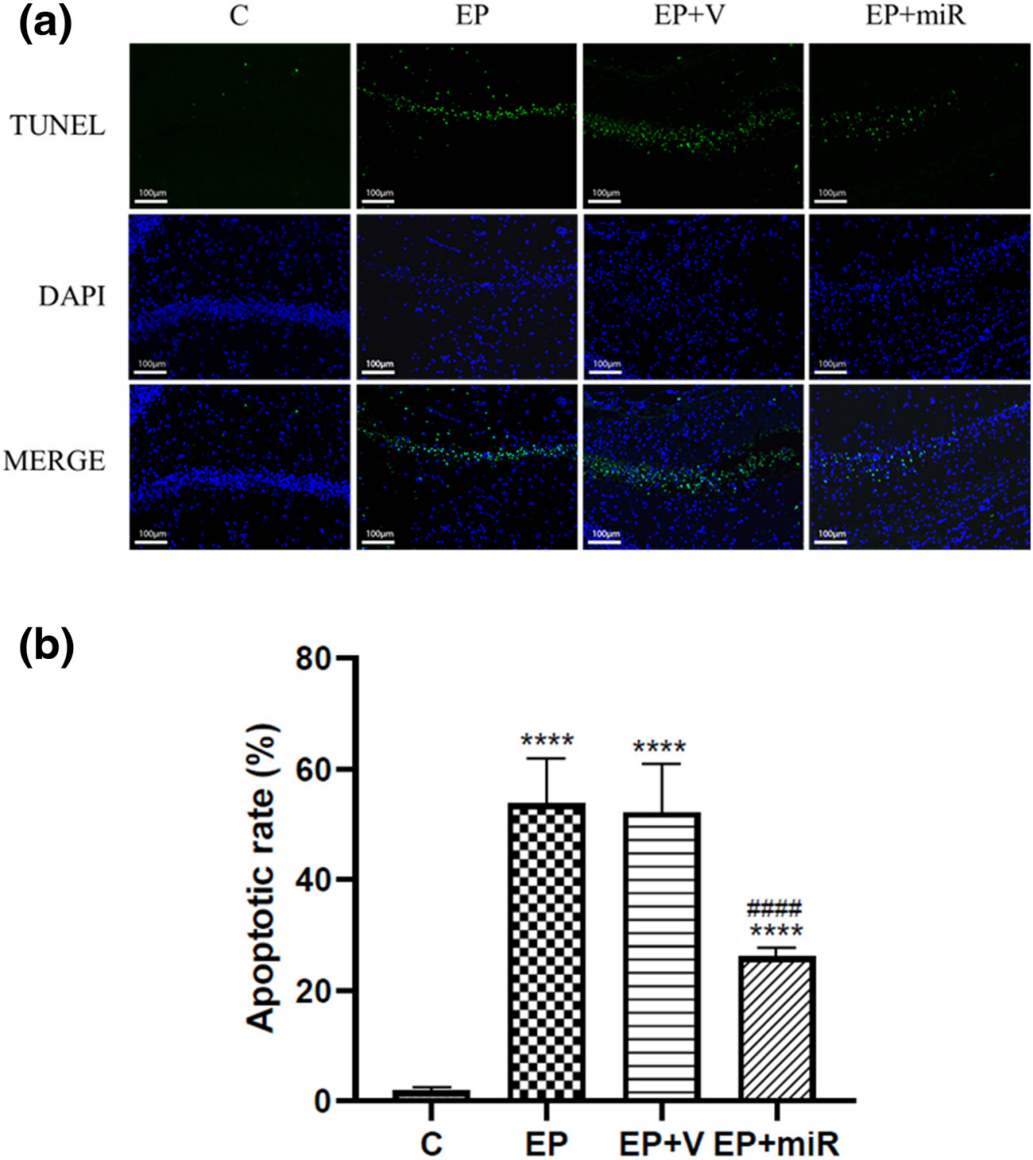
Exogenous miR-322-5p prohibited epilepsy-induced neuronal apoptosis. Notes: (a) apoptosis detected by Tunel. Magnification 200×. (b) Quantitative data of the apoptosis. ****p < 0.0001 compared with control; ####*p* < 0.0001 compared with EP + V group. Abbreviations: C: control, EP: epilepsy, EP + V: epilepsy + vehicle, EP + miR: epilepsy + miR-322-5p intervention group.

Compared with the control group, apoptotic cells in the hippocampal CA1 area of the Ep group increased significantly (Figure 5). It can be seen that the epilepsy model triggers typical apoptosis in the CA1 area. Compared with the EP + V group, the apoptotic cells in the hippocampal CA1 area of the EP + miR group were significantly reduced (Figure 5). It can be concluded that upregulation of miR-322-5p can effectively inhibit the apoptosis of hippocampal neurons in the CA1 region of the hippocampus of rats.

## 4 Discussion

The occurrence and development of epilepsy are potentially related to inflammation. The inflammatory mediators directly affect the function of neurons and glial cells, increasing the excitability of the nervous system. The epileptic seizures can also promote the neuroinflammatory response and apoptosis, forming the pathological basis of SE and refractory epilepsy [12]. However, the signaling network underlying neuroinflammation in epilepsy remains to be explored.

The TLR4/NF-κB signaling pathway is responsible for inflammatory responses in different disorders, including epilepsy [13]. In the current study, we found that the TLR4/TRAF6/NF-κB signaling pathway was upregulated in the brain of SE rats. Previous studies demonstrated that different inflammatory mediators, including tumor necrosis factor (TNF)-α, IL-1β, and IL-6, may contribute to epilepsy development and progression. Of note, an elevated expression of TLR4 was identified in surgical specimens of drug-resistant temporal lobe epilepsy, focal cortical dysplasia, and tuberous sclerosis patients [14]. The inhibition of HMGB1/TLR4 was demonstrated to reduce the incidence of seizures [15], while a downstream effector of TLR4, TRAF6, played an essential role in Th1 inflammation [16]. In addition, the inhibition of TLR4/NF-κB signaling pathway and IL-1β in the hippocampus attenuated the severity of SE in rats [17]. Overall, these findings suggest that the TLR4/NF-κB signaling pathway may have an important role in the development of epilepsy.

Here, we demonstrated another potential regulatory mechanism by which TLR4 signaling was amplified in the SE rat brains. miR-322-5p was one of the most reduced microRNA species after the SE establishment in our study as well as in another study [18]. Our results showed that miR-322-5p downregulated TRAF6, which activated the NF-κB signaling pathway. A previous study indirectly supported our notion where an increased level of miR-322-5p elevated the EZH2 and activated Akt/GSK3β pathway, thereby protecting myocardial cells from ischemic reperfusion injury [19]. On the contrary, in the SE rat brain, miR-322-5p was significantly reduced and associated with a higher level of neuroinflammation.

Compared with the control group, rats injected with exogenous miR-322-5p after SE induction showed a significant decrease in the expression of TLR4, TRAF6, NF-κB and IL-1β, and an increase in the expression of GABA. A previous study found that miR-322 mimics decrease the expression levels of NF-κB1 (p50) and inflammatory cytokines in LPS-stimulated murine macrophages [20]. This finding suggested the feasibility of exogenous miR-322-5p as an inhibitor of neuroinflammation. Emerging evidence demonstrates that stereotactic injection or antagomirs or mimic molecules into the hippocampal regions not only is feasible but also reduces the incidence of seizures in rodent models [21]. Thus, our findings provided support for injecting exogenous miR-322-5p as a preventive measure for reducing chronic neuroinflammatory circuits, namely, the TLR4/TRAF6/NF-κB pathway.

Apoptosis is an important form of neuronal damage after SE. After SE, the release of excitatory amino acids increases intracellular calcium overload, caspase cascades, promote oxidative stress, and caspase-3 activation, and then induce cell apoptosis [22]. We confirmed that epileptic seizures induce apoptosis of hippocampal neurons, and injection of exogenous miR-322-5p can reduce cell apoptosis caused by epilepsy. Studies have found that miR-322 has the effect of reducing cell apoptosis, but its mechanism of action is still unclear [23]. miR-322 regulated the apoptosis of neural stem cells through silencing NADPH oxidase 4, thereby inhibiting the occurrence of neural tube defects in rats [24]. miR-322 can regulate breast cancer cell apoptosis by targeting NF-κB1 [25]. Our experiments have found that exogenous miR-322-5p can reduce neuronal cell apoptosis after SE, accompanied by downregulation of TLR4, TRAF6, and NF-κB expression. It is speculated that the mechanism of miR-322 inhibiting apoptosis may be related to the TLR4/TRAF6/NF-κB pathway, but it still needs more in-depth experiments to verify.

## 5 Conclusion

The present study provided evidence that the TLR4/TRAF6/NF-κB signaling pathway is positively associated with neuroinflammation and apoptosis in pilocarpine-induced epilepsy. The miR-322-5p is a negative regulator of epileptic seizures and may serve as a potential medication for epileptic seizures. Further investigation is warranted to test this hypothesis.

## Abbreviations

GABA: γ-aminobutyric acid
GAPDH: glyceraldehyde 3-phosphate dehydrogenase
IL-1β: interleukin-1β
NF-kB: nuclear factor-k-gene binding
TLR4: Toll-like receptor 4
TRAF6: tumor necrosis factor receptor-associated factor 6
